# Leveraging Diverse Cell-Death Patterns to Decipher the Interactive Relation of Unfavorable Outcome and Tumor Microenvironment in Breast Cancer

**DOI:** 10.3390/bioengineering12040420

**Published:** 2025-04-15

**Authors:** Yue Li, Ting Ding, Tong Zhang, Shuangyu Liu, Jinhua Wang, Xiaoyan Zhou, Zeqi Guo, Qian He, Shuqun Zhang

**Affiliations:** 1 Department of Clinical Laboratories, The Second Affiliated Hospital of Xi’an Jiaotong University, Xi’an 710004, China; liyuelywwcx@126.com (Y.L.); dingting1994@163.com (T.D.); sxycgzq@126.com (Z.G.); qianh0511@126.com (Q.H.); 2 Department of Oncology, The Second Affiliated Hospital of Xi’an Jiaotong University, Xi’an 710004, China

**Keywords:** breast cancer, tumor prognosis, new neoplasm occurrence, programmed cell death, machine learning, immune microenvironment

## Abstract

Background: Programmed cell death (PCD) dynamically influences breast cancer (BC) prognosis through interactions with the tumor microenvironment (TME). We investigated 13 PCD patterns to decipher their prognostic impact and mechanistic links to TME-driven outcomes. Our study aimed to explore the complex mechanisms underlying these interactions and establish a prognostic prediction model for breast cancer. Methods: Using TCGA and METABRIC datasets, we integrated single-sample gene set enrichment analysis (ssGSEA), weighted gene co-expression network analysis (WGCNA), and Least Absolute Shrinkage and Selection Operator (LASSO) to explore PCD-TME interactions. Multi-dimensional analyses included immune infiltration, genomic heterogeneity, and functional pathway enrichment. Results: Our results indicated that high apoptosis and pyroptosis activity, along with low autophagy, correlated with favorable prognosis, which was driven by enhanced anti-tumor immunity, including more M1 macrophage polarization and activated CD8+ T cells in TME. PCD-related genes could promote tumor metastasis and poor prognosis via VEGF/HIF-1/MAPK signaling and immune response, including Th1/Th2 cell differentiation, while new tumor event occurrences (metastasis/secondary cancers) were linked to specific clinical features and gene mutation spectrums, including TP53/CDH1 mutations and genomic instability. We constructed a six-gene LASSO model (*BCAP31*, *BMF*, *GLUL*, *NFKBIA*, *PARP3*, *PROM2*) to predict prognosis and identify high-risk BC patients (for five-year survival, AUC = 0.76 in TCGA; 0.74 in METABRIC). Therein, the high-risk subtype patients demonstrated a poorer prognosis, also characterized by lower microenvironment matrix and downregulated immunocyte infiltration. These six gene signatures also showed prognostic value with significant differential expression in gene and protein levels of BC samples. Conclusion: Our study provided a comprehensive landscape of the cancer survival difference and related PCD-TME interaction axis and highlighted that high-apoptosis/pyroptosis states caused favorable prognosis, underlying mechanisms closely related with the TME where anti-tumor immunity would be beneficial for patient prognosis. These findings highlighted the model’s potential for risk stratification in BC.

## 1. Introduction

Breast cancer (BC) is one of the most common malignant tumors affecting women worldwide, with a high mortality rate. In recent years, with the advancement of chemotherapy and radiation therapy, the long-term survival and cure rates of BC patients have greatly improved. However, some BC patients, including triple-negative breast cancer (TNBC) patients, displayed high-grade, aggressive tumors with a high rate of distant metastasis and poorer prognosis [1,2]. BC would show a poor prognosis when it spread to lymph nodes or distant organs (metastatic tumor) or triggered second primary cancers (SPCs), leading to increased difficulties for treatment and reduced life quality for patients. Compared to primary BC, SPC was the secondary tumor that appears in the human body after excluding tumor metastasis. The new tumor occurrence, including metastatic or secondary tumors in breast cancer patients would greatly affect the prognosis. However, there was not enough attention paid to SPC closely associated with the long-term complications of anticancer drugs and radiation and not enough breakthroughs made in the distant metastasis, and thus further exploration and improvement were needed urgently. In general, due to the large and heterogeneous BC population, the development mechanism of BC with unfavorable prognosis continues to pose significant challenges; the exploration of novel therapeutic strategies inspired by mechanism research are needed to improve the management of BC patients.

The tumor microenvironment (TME), a dynamic network of extracellular matrix components and immune cells, plays a crucial role in the progression and metastasis of cancer by modulating cellular homeostasis and influencing tumor behavior [3]. Programmed cell death (PCD) mechanisms have emerged as critical regulators of tumor immunogenicity and therapeutic response [4]. PCD is a fundamental process in maintaining tissue homeostasis, and dysregulation of PCD pathways has been implicated in cancer development. Apoptosis, necroptosis, pyroptosis, ferroptosis, cuproptosis, entotic cell death, netotic cell death, parthanatos, lysosome-dependent cell death, autophagy-dependent cell death, alkaliptosis, oxeiptosis, and disulfidptosis are different modes of PCD that have been identified in recent years, which have been discovered to play crucial roles in modulating the immunosuppressive TME and determining clinical outcomes of the cancer therapeutic approaches [5]. For example, inhibition of apoptosis has been associated with tumor progression and resistance to chemotherapy. On the other hand, induction of ferroptosis has been shown to suppress tumor growth in breast cancer models. These pathways exhibit distinct mechanisms, and prior studies have focused on single PCD pathway; the interactive impacts of diverse PCD patterns on TME-driven prognosis remains unclear.

Some studies began to highlighted the intricate relationship between the TME and PCD in breast cancer. PCDs are dynamically plastic, exert a dual role in distinct contexts and stages of cancer development, and some studies indicated that the effect of PCDs were regulated by the tumor microenvironment. The game between pro-survival and pro-death pathways regulated by PCD, including pyroptosis or autophagy, shapes the heterogeneity and complexity of the tumor immunity in TMEs. Ershaid et al. confirmed that pyroptosis induced by NLRP3 and IL-1β secretion might adjust the TME towards an immune suppressive microenvironment, promoting cancer proliferation and invasion in mouse and human breast cancer [6,7]. Hage et al. demonstrated that sorafenib induced caspase-1-related macrophage pyroptosis and thus caused the liberation of the cytokines and enhanced the cytotoxicity of NK cytotoxicity for the efficient tumor cell killing [8]. Moreover, Hypoxic conditions within the TME have been found to induce apoptosis resistance in breast cancer cells. Similarly, the TME can modulate the sensitivity of breast cancer cells to ferroptosis, a form of iron-dependent cell death, thereby influencing the efficacy of ferroptosis-inducing therapies. Despite these advancements, our understanding of the complex interactive regulation of TME and various PCD patterns in breast cancer remains limited. We aimed to characterize the landscapes of PCD-associated genes within the TME, and investigate the mechanisms linking these PCD/TME-associated landscapes to unfavorable clinical outcomes in BC, to establish a BC prognosis prediction or risk stratification model using machine learning methods. This research will provide novel insights of breast cancer biology and pave the way for the development of novel therapeutic strategies targeting the TME and PCD.

## 2. Materials and Methods

### 2.1. Data Source

**Figure 1:**
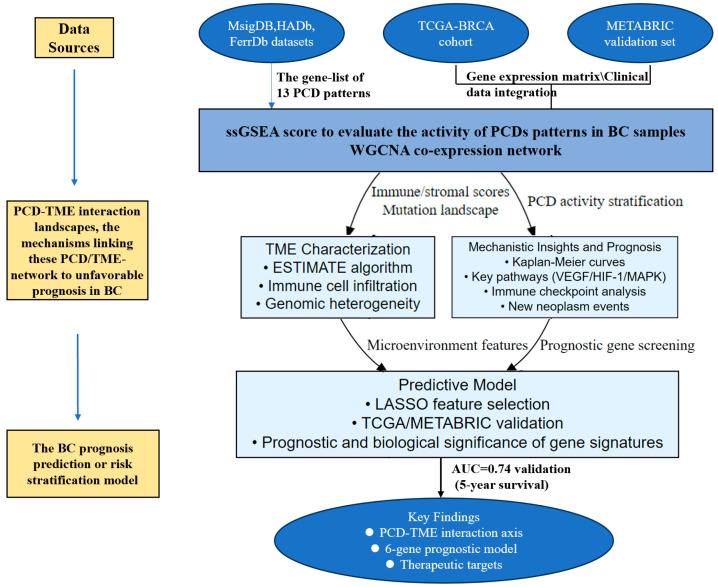
Study design overview.

The PCD-related genes contained the key regulatory genes of 13 PCD patterns, including apoptosis, necroptosis, pyroptosis, ferroptosis, cuproptosis, entotic cell death, netotic cell death, parthanatos, lysosome-dependent cell death, autophagy-dependent cell death, alkaliptosis, and oxeiptosis, which were acquired from Molecular Signatures Database (MsigDB, https://www.gsea-msigdb.org/gsea/msigdb/ (accessed on 18 April 2024)), Human Autophagy Database (HADb, https://autophagy.lu/, accessed on 18 April 2024), FerrDb [9] (http://www.zhounan.org/ferrdb/current/, accessed on 18 April 2024), and related studies [10,11]. Herein, as shown in Supplementary Table S1, 622 apoptosis-related genes (including extrinsic and intrinsic apoptotic pathway), 204 necroptosis genes [12], 57 pyroptosis genes, 502 ferroptosis genes, 62 cuproptosis genes, 512 autophagy genes, 16 entotic cell death genes, 10 parthanatos genes, 10 netotic cell death genes, 7 alkaliptosis genes, 209 lysosome-dependent cell death genes, 21 oxeiptosis genes, and 15 disulfidptosis genes were collected. And so, a total of 1699 PCD-related genes from RNA-seq were brought into the analysis. Gene expression datasets in our study and corresponding clinical information were obtained from The Cancer Genome Atlas (TCGA), Molecular Taxonomy of Breast Cancer International Consortium (METABRIC). The gene and protein expression data in this study were also acquired from GEPIA (gepia.cancer-pku.cn/, accessed on 15 October 2024) and Human Protein Atlas (https://www.proteinatlas.org/, accessed on 20 October 2024), visualized TCGA dataset platforms, which involved RNA sequencing expression data and antibody-based TCGA cancer proteome, respectively. The flow chart of the study design and some findings were shown in Figure 1.

### 2.2. The Single-Sample Gene Set Enrichment Analysis (ssGSEA) Algorithm

ssGSEA algorithm, a computational method based on gene set enrichment analysis, was applied to estimate the abundance of gene signatures for 13 PCD patterns. The ssGSEA score of each breast cancer sample was calculated by inputting each PCDs-related gene list (Table S1), which could be used to analyze and evaluate each PCD pattern activity of breast cancer, as used in our previous autophagy-related study [13].

### 2.3. Tumor Microenvironment (TME) and Immunocyte Infiltration Analysis

Immune and stromal components were quantified using the ESTIMATE algorithm in R (v4.3.1), a conventional method used in many studies [9,10], with scores reflecting TME composition: the immune infiltration (ImmuneScore), overall stromal content (StromalScore), and the sum of the two components (EstimateScore), by which their higher scores indicated larger ratios of the respective component. The ssGSEA in the “GSVA” R package was used to analyze immune infiltration in BC by analyzing the distribution of 28 immune cells.

### 2.4. Weighted Gene Co-Expression Network Analysis (WGCNA)

We used consensus WGCNA (via R 4.3.1) to cluster genes with similar expression patterns and identify those co-expressed gene modules linked to cancer prognosis (between the dead and alive BC patients). The total of 1699 PCD-related genes were inputted and screened for network constructions. Based on the criterion of approximate scale-free topology, we chose the appropriate soft thresholding power (β) in the use of the function pickSoftThreshold, to which co-expression similarity is raised to calculate adjacency. The cluster dendrogram completed a clustering analysis of genes with similar expression patterns, compiling them into co-expression modules, and thus 7 kinds of colors (modules) were identified, and among them, the gray module was usually considered as the unassigned-genes module. Identifying consensus modules significantly correlated with clinical traits in different survival states and, in the same direction (positive or negative correlation) as well, could help recognize interesting modules as well as with high significance for clinical traits, including overall survival (OS), age of initial diagnosis, TNM and stages, lymph node ratio (LNR), positive margin, estrogen receptor (ER), progesterone receptor (PR), human epidermal growth factor receptor-2 (HER2), and new neoplasm event.

### 2.5. Enrichment Analysis for Biological Function and Pathway

Module biological function was analyzed using g:Profiler (https://biit.cs.ut.ee/gprofiler, accessed on 27 August 2024), including gene ontology (GO), Kyoto Encyclopedia of Genes and Genomes (KEGG) pathway, Reactomen, and WikiPathways. Enrichments were considered significant when the adjusted *p* value was <0.05.

### 2.6. Identification of Differential Gene Expression (DEGs)

Expression profile data of the groups analyzed in this study (Metastasis vs. Control, New Primary Tumor vs. control) were used to perform the identification of DEGs using R package, EdgeR for examining DEGs of RNA-Seq count data. DEGs were considered significant based on the following criteria: |log2 fold change (FC)| > 1; both the *p*-value and FDR < 0.05.

### 2.7. Survival Analysis

For survival analysis, the overall survivals (OS) of cancer patients with a follow-up time greater than 30 days could be analyzed using Kaplan–Meier estimations. In addition, the influence of PCD patterns for progression-free survival (PFS) and disease specific survival (DSS) in the cases we studied was also analyzed. The different groups of patients were evaluated for their survival differences; which is to say, we could divide patients into high/low expression groups based on various factors, such as gene expression values or model risk scores in our study. The hazard ratio (HR) was determined via a Cox regression model. The survival analysis was considered a significant difference based on log rank test *p* < 0.05.

### 2.8. Gene Mutation Landscapes

Gene mutation landscape and tumor genomic heterogeneity analysis, including tumor mutational burden (TMB) and loss of heterozygosity (LOH) in all BC types, were performed based on the maftools R package with TCGA tumor exon sequencing data and online platforms for TCGA patient data based on R, including the cBioPortal database (https://www.cbioportal.org/, accessed on 3 September 2024) and SangerBox database (http://www.sangerbox.com/, accessed on 3 September 2024).

### 2.9. Least Absolute Shrinkage and Selection Operator (LASSO)

LASSO is a linear regression method based on L1 regularization, which is better used for variable selection. In this study, we used the R software package glmnet to integrate OS, survival status, and gene expression profiles and conducted a regression analysis using a Lasso–Cox method. In addition, we also set a 10-fold cross validation to obtain the optimal model. By setting the Lambda value to 0.025785075482234, this study obtained the initial risk model formula of 24 genes, to evaluate and predict the survival risk of patients. Through further screening, six genes were identified for the optimal PCDs-risk model by 10-fold cross validation and setting the Lambda value to 0.00549078283426211, and based on this, the PCD risk scores for each BC patients were calculated. We used time-dependent receiver-operating characteristic (ROC) and Kaplan–Meier survival curves to evaluate the performance of the model.

### 2.10. Analysis of Affected Tumor-Related Pathways and Therapeutic Targets

Furthermore, based on the gene signatures from the LASSO model, we analyzed potential effects of gene mRNA on cancer-related pathway activity, utilizing Gene Set Cancer Analysis (GSCA) platform (https://guolab.wchscu.cn/GSCA/#/, accessed on 7 March 2025). The core analysis principle is to use Gene Set Variation Analysis (GSVA) to evaluate the differences in pathway activity (including apoptosis, cell cycle, DNA damage, epithelial–mesenchymal transition, Hormone AR, Hormone ER, PI3K/AKT, RAS/MAPK, RTK, TSC/mTOR pathways) between high and low gene mRNA expression [11,12]. The drugs targeting gene signatures of the LASSO model were obtained through Comparative Toxicogenomics Database (CTD, http://ctdbase.org/, accessed on 9 March 2025) and DrugBank database.

### 2.11. Statistical Analysis

Statistical significance was determined as *p* < 0.05 and performed with GraphPad Prism 9 and SPSS version 21.0. Continuous variables were presented as mean ± standard deviation (SD). The results of two groups were analyzed using Student’s *t* test, three or more groups were compared using one-way analysis of variance (ANOVA) test, and further difference between the groups was analyzed with the Dunnett method. To analyze the associations between categorical variables, a Chi-square test or Fisher’s exact test was performed, depending on the sample size and expected frequencies.

## 3. Results

### 3.1. Prognostic Difference in Breast Cancer Affected by Expression States of 13 PCD Patterns

**Figure 2:**
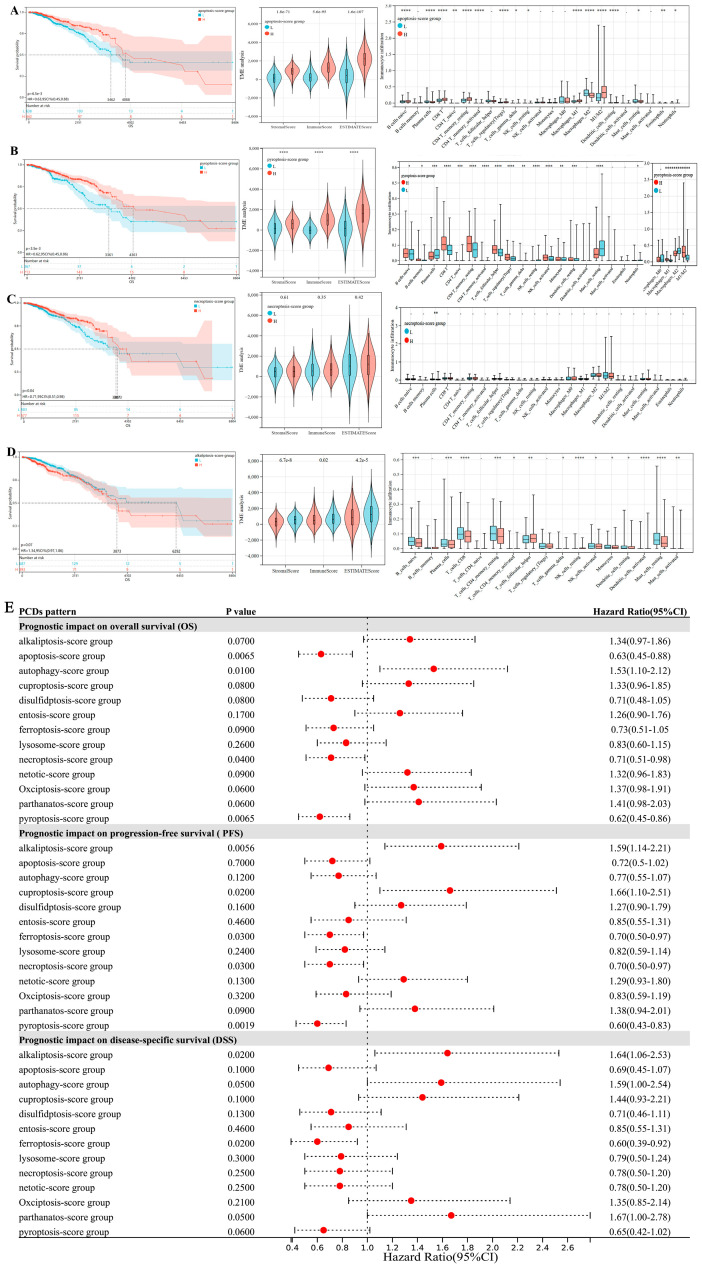
Prognostic differences and tumor microenvironment characteristics of breast cancer subtypes based on PCD activity scores. (**A**–**D**) The prognostic influence of the apoptosis/pyroptosis/necroptosis/alkaliptosis-score subtypes in breast cancer and the corresponding PCD-TME interaction relationship. (**E**) The forest plot based on the survival analysis: the influence for overall survival (OS), progression-free survival (PFS), and disease-specific survival (DSS) between the two groups with high and low PCDs score in the cases we studied (complete Kaplan–Meier curves in Supplementary Figure S1). (* *p* < 0.05, ** *p* < 0.01; *** *p* < 0.001, **** *p* < 0.0001).

Using ssGSEA, we evaluated the activity of diverse PCD patterns and classified BC patients into score-high/low subtypes (e.g., apoptosis-score high and low, etc.) The survival analysis revealed significant associations between PCD activity scores and clinical outcomes in BC. High-apoptosis (HR = 0.63, 95% CI 0.45–0.88, *p* < 0.01), high-pyroptosis (HR = 0.62, 95% CI 0.45–0.86, *p* < 0.01), and high-necroptosis (HR = 0.71, 95% CI 0.51–0.98, *p* = 0.04) scores correlated with more prolonged OS in BC, whereas low-autophagy activity predicted significantly favorable prognosis (Figure 2A–E). In addition, the activities of alkaliptosis, ferroptosis, disulfidptosis, oxciptosis, parthanatos, cuproptosis, and netotic cell death were marginally relevant with survival outcomes in BC patients, but no significant prognostic impacts of entosis/lysosome cell death (Figure 2E, all Kaplan–Meier curves in Supplementary Figure S1). These results indicated the important influence of the PCD pattern activity for the progression and prognosis of BC.

Furthermore, high-apoptosis/pyroptosis activity correlated with immune activation, evidenced by elevated ImmuneScore, StromalScore, CD8+ T cells, T follicular helper (Tfh)/regulatory T (Treg) cells, and M1 macrophage polarization (tumor-associated macrophages M1/M2). Meanwhile, in our previous research [13], the autophagy score-low subtypes displayed the more favorable prognosis compared with score-high, associated with their immune-activated features, which manifested as high CD8+T, Tfh, Treg, NK cells, and tumor-associated macrophages M1/M2. Therefore, these results indicated that high-apoptosis/pyroptosis and low-autophagy states could all improve the prognosis of BC via the more anti-tumor immune responses associated with the tumor microenvironment, which is specifically mainly achieved through promoting macrophage polarization towards anti-tumorigenic M1 macrophages and CD8+ T cells.

### 3.2. Co-Expression Networks Link PCD Gene Modules to Clinical Features in Breast Cancer

**Figure 3:**
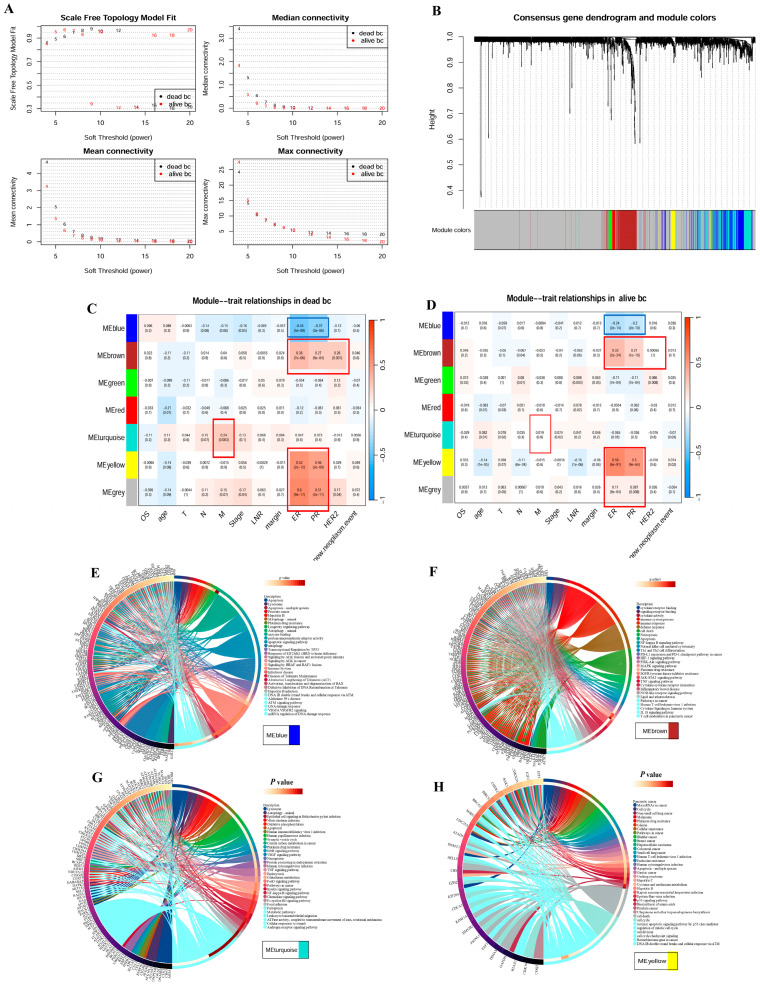
Identification of hub genes by WGCNA between the dead and alive BC patients. (**A**) Scale independence and mean connectivity of β from 1 to 30. (**B**) The cluster dendrogram. (**C**,**D**) Heatmap of the consensus modules identification for dead (**C**) and alive patients (**D**) and theirs correlations with clinical traits, for example, TNM stages, ER (-)/PR(-)/HER2(-), new neoplasm event (metastatic tumor or new primary tumor during follow-up). (**E**–**H**) The top GO enrichment terms and KEGG enrichment pathways of genes from MEblue (**E**), MEbrown (**F**), MEturquoise (**G**), and MEyellow (**H**).

Based on the gene expression profiles of 13 PCD patterns, the gene co-expression modules construction of different survival outcomes (Death, Figure 3C, N = 148; Alive, Figure 2D, N = 940), and the correlations between module eigengenes and clinical features were displayed in Figure 3A–D. Since the idea of consensus WGCNA was to identify specific or universal co-expression gene modules associated with clinical features in BC patients with different prognosis, only those genes with the consistent co-expression in both dead and alive patients could be classified into the same module, and thus a large number of genes were classified into the gray module (unassigned genes module). Consensus WGCNA identified that ER/PR(-) status was negatively correlated with the blue module (Meblue) and positively correlated with MEbrown/Meyellow. The turquoise module had the significantly positive correlation with tumor M stage (Tumor metastasis occurring) but lacked an obvious correlation in alive patients, suggesting that these genes from the turquoise module could explain the specificity of the tumor metastasis mechanism in dead patients and further relate to the unfavorable prognosis of BC. These above modules were identified as clinically significant modules for further analysis. In addition to several PCD pathways, such as apoptosis, autophagy, necroptosis, and ferroptosis, the blue module was also enriched in TP53 transcriptional regulation, immune system, ALK signaling in cancer, DNA damage response, and VEGFA/VEGFR2 signaling (Figure 3E). The brown module was also enriched in NF-κB signaling pathways, many processes of immune response (natural-killer-cell-mediated cytotoxicity, Th1 and Th2 cell differentiation, PD-L1 expression and PD-1 checkpoint pathways in cancer, cytokine signaling, IL18 signaling pathways, and T cell modulation in pancreatic cancer, HIF-1 signaling, PI3K-Akt signaling, MAPK pathways, and JAK-STAT pathways (Figure 3F)). The turquoise module was also enriched in response to virus (epithelial cell signaling in helicobacter pylori infection, human papillomavirus infection), central carbon metabolism in cancer, ErbB signaling, VEGF signaling, TNF signaling, Endocytosis, Insulin signaling, NF-κB pathway, chemokine signaling, focal adhesion, leukocyte transendothelial migration, and androgen receptor pathways (Figure 3G). The yellow module was enriched in cell cycle, cell division, cell cycle checkpoint signaling, p53 signaling, many cancer pathways, and several responses to virus (Figure 3H). Platinum drug resistance was associated with the four modules.

### 3.3. Immune Microenvironment Patterns with Different Survival Outcomes in Breast Cancer

**Figure 4:**
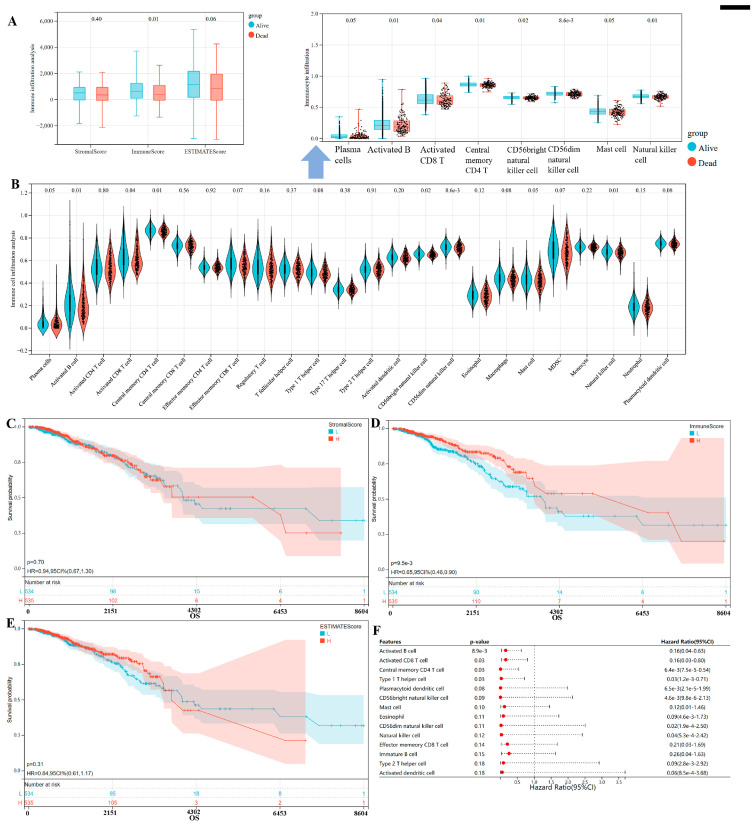
Identification of immune microenvironment patterns between the dead and alive BC patients. (**A**,**B**) The TME in the alive and dead BC patients. (**C**–**E**) The survival analysis of StromalScore (**C**), ImmuneScore (**D**), and ESTIMATEScore (**E**) for BC through the separation of BC patients into two clusters based on the median score. (**F**) The prognosis influence of all kinds of immunocytes for BC.

A high StromalScore or ImmuneScore indicates more stromal or immune components in TME, and ESTIMATEScore is the total sum of the StromalScore and ImmuneScore, representing the combined ratio of two components in TME. We could observe the higher ImmuneScore in the alive BC than the dead patients (Figure 4A), and meanwhile the higher immune cell infiltration levels in the patients with good outcomes, manifested as the increased plasma cells, activated B cells, activated CD8 T cells, central memory CD4 T cells, CD56bright natural killer cells, CD56dim natural killer cells, and mast cells (Figure 3B), suggesting the important roles of those for BC survival outcomes associated with the anti-tumor immune response. Meanwhile, as shown in Figure 4C–E, our results show only a lower risk of death in the high ImmuneScore (HR = 0.65, 95% CI 0.46–0.90, log rank *p* = 0.0095). Considering the significantly higher ImmuneScore in the alive BC, while no significant difference was between the StromalScore/ESTIMATEScore and prognosis, these results all suggested the favorable prognosis of BC depended more on higher immune cell infiltration in TME. Furthermore, among all kinds of immunocytes, the existing evidence supports the crucial role of activated B cell, activated CD8+ T cell, central memory CD4+ T cell and type 1 helper cell for BC prognosis associated with the anti-tumor immune surveillance and regulation (Figure 4F).

### 3.4. The Correlations of Cell-Death Patterns (Apoptosis, Pyroptosis) and Immune Checkpoint Gene Expression

**Figure 5:**
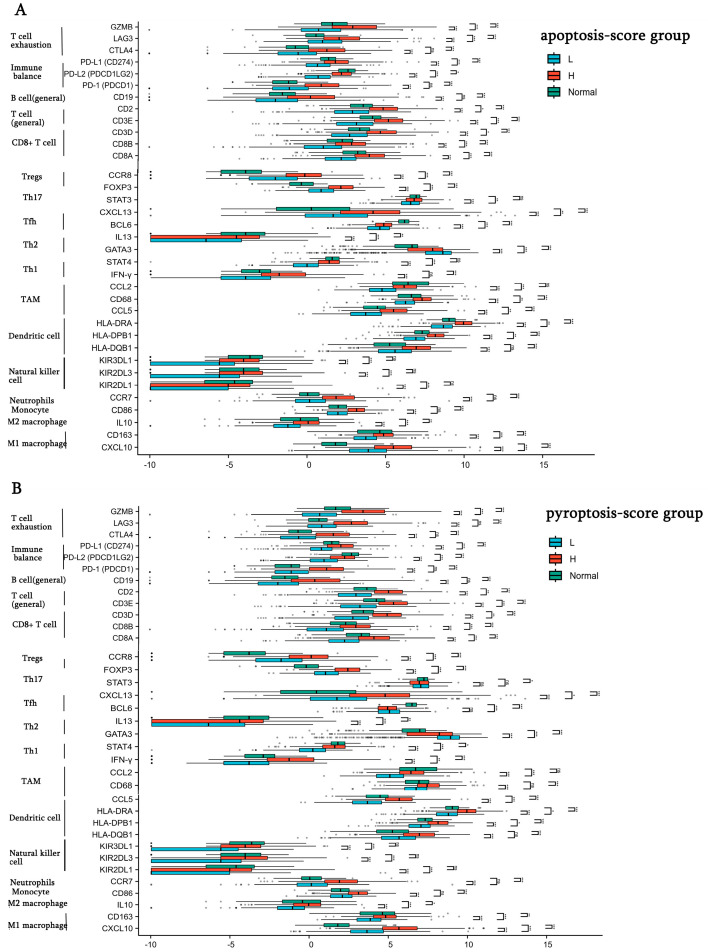
The differences in immune checkpoint expression levels among the different apoptosis/pyroptosis activity BC subtypes. (**A**) The differences in immune checkpoint expression levels in the low-apoptosis, high-apoptosis BC subtypes and normal tissues. (**B**) The differences in immune checkpoint expression levels in the low-pyroptosis, high-pyroptosis BC subtypes and normal tissues. (* *p* < 0.05, ** *p* < 0.01; *** *p* < 0.001, ns, no statistical difference).

As mentioned above, our findings indicated that the different apoptosis/pyroptosis activity of BC caused the significantly different prognosis, which is closely related to its immune microenvironment. In Figure 5A,B, we further observed elevated expression of many immune checkpoints in the BC subtype with the high-apoptosis/pyroptosis activity, such as PD-1, PD-L1, CCR8, CXCL13. However, this trend was reversed in the low PCD-activity subtypes. The BC with low apoptosis/pyroptosis activity might lead to poor immune checkpoint therapy and further exacerbate poor prognosis.

### 3.5. New Tumor Occurrence Caused Poor Prognosis of Breast Cancer Patients

**Figure 6:**
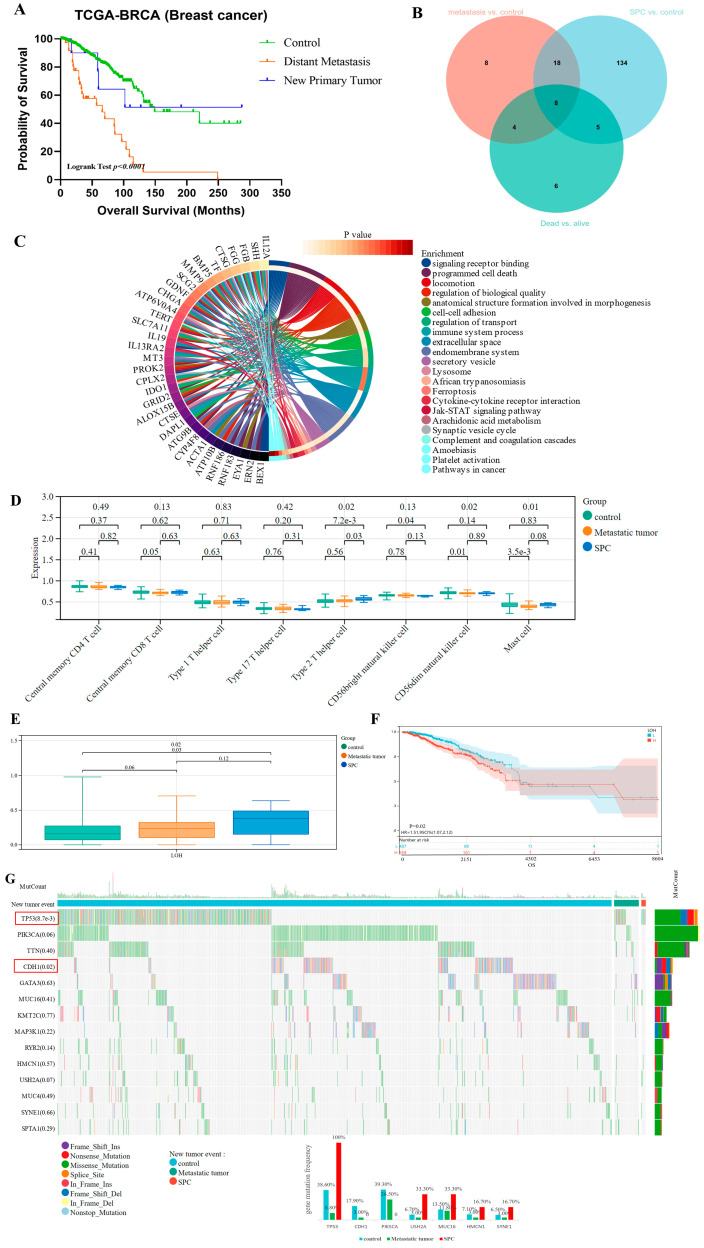
The landscape analysis of new tumor occurrence associated with the poor prognosis of breast cancer. (**A**) The prognostic impact of new tumor occurrence during follow-up for breast cancer, through the Kaplan–Meier method. (**B**) The Venn plot of the DEGs in the three comparison groups: between metastatic tumor and control, SPC and control, and dead and alive BC patients. (**C**) The GO enrichment terms and KEGG enrichment pathways of overlapping genes from (**B**). (**D**) The comparison of immune cell infiltration levels. (**E**,**F**) Tumor genomic heterogeneity analysis, LOH, in the new tumor event. (**G**) The frequently mutated genes of breast cancer. Gene mutation landscape associated with new tumor event in BC.

Furthermore, we analyzed the clinical characteristics of BC patients with new tumor events (distant metastasis, and new primary tumor/or second primary cancer, SPC) during follow-up (Table 1). We could observe the significantly poorer prognosis in patients with new tumor occurrence during follow-up, compared with the other BC patients without them (Figure 6A). The higher negative status of ER/PR/HER2 was observed in BC patients with new tumor events, especially a higher rate of triple-negative breast cancer (TNBC) in SPCs. We did not observe a significant impact of apoptosis/pyroptosis/autophagy activity on the development of new tumor occurrence (Chi square, *p* > 0.05).

The DEGs were analyzed in the three comparison groups: between metastatic tumor and control, SPC and control, and dead and alive BC patients, and a total of 35 overlapping genes were screened (Figure 6B); further combining module genes, 24 genes were obtained for biological enrichment analysis. Cytokine–cytokine receptor interaction, cell–cell adhesion, regulation of transport, immune system process, secretory vesicle, lysosome, ferroptosis, Jak-STAT signaling pathway, synaptic vesicle cycle, complement and coagulation cascades, and platelet activation were significantly enriched (Figure 6C). The difference in the immune microenvironment is not obvious in patients with new tumor occurrence (Figure 6D), but these patients displayed the significantly increased LOH (high LOH was an unfavorable factor for BC prognosis, Figure 6E,F). Moreover, the specific genetic mutation patterns were shown in three groups, including the obvious changes in mutation frequency in *TP53*, *CDH1*, *PIK3CA*, and *USH2A* (Figure 6G).

### 3.6. Identification of Markers Predicting the Prognosis of Breast Cancer

**Figure 7:**
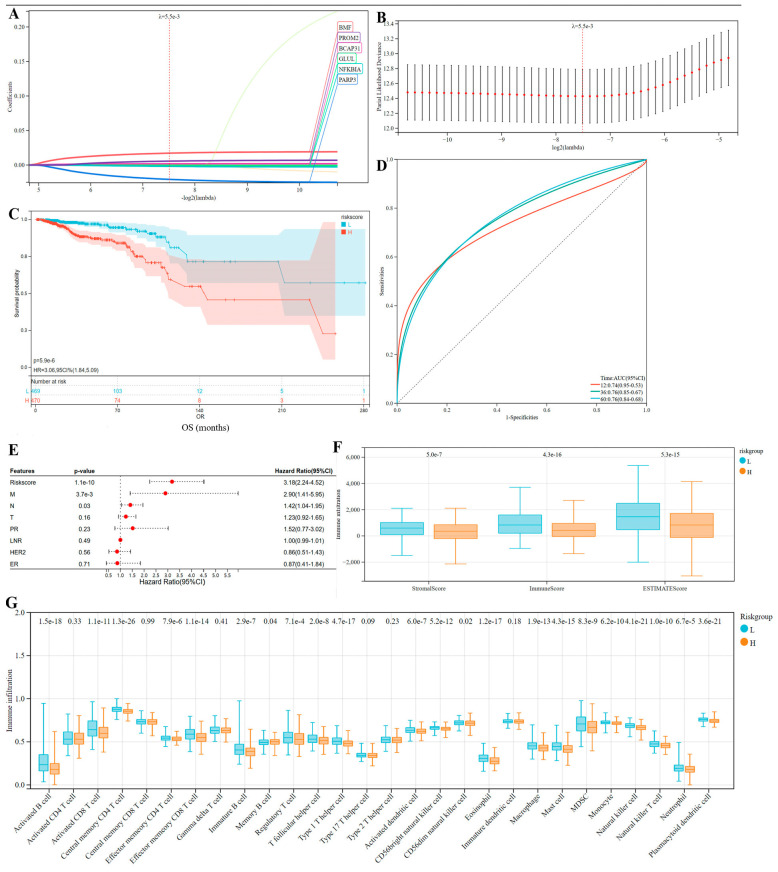
Construction of the PCDs-related prognostic model based on the prognostic PCD-related genes. (**A**,**B**) LASSO analysis; (**C**) K-M analysis of PCDs-related risk model; (**D**) ROC curve of the risk model with significant prognostic prediction value analyzed based on all TCGA-BCs patients; (**E**) univariate Cox analysis; (**F**,**G**) anti-tumor immunity between the two PCDs risk subtypes; and comparison of (**F**) immune score/stromal score/ESTIMATE score and (**G**) immune cell infiltration fraction among the two PCDs risk subtypes.

From 1699 PCD-related genes (especially, the overlapping DEGs of tumor occurrence), LASSO regression screened six genes for the optimal PCDs risk model using a 10-fold cross validation and setting the Lambda value to 0.00549078283426211. Also, the risk scores for each BC patient was calculated (Figure 7A,B): RiskScore = 0.00177439035044029 × *BCAP31* expression + 0.0172022499888911 × *BMF* expression − 0.000487210250283775 × *GLUL* expression − 0.00179314430415852 × *NFKBIA* expression − 0.0207439318347913 × *PARP3* expression + 0.00566437326914523 × *PROM2* expression.

As shown in Figure 7C, the group with high-risk scores displayed the significant unfavorable prognosis than the low-risk group based on the median risk score that could divide BC patients into two groups. Also, the ROC curve was utilized to assess the projected accuracy of this initial model, and the AUC separately under one-year, three-year, and five-year survival curves showed good prognostic efficacy (AUC > 0.75) (Figure 7D). Univariate Cox analysis demonstrated that M/N stage and risk scores were closely related to BCs prognosis (Figure 7E), suggesting that PCD risk scores would be an important risk factor indicating the prognosis of breast cancer. Moreover, significant differences were observed in the TME between the high-risk and low-risk patients divided by this model (Figure 7F,G). The high-risk group showed lower microenvironment matrix scores and immune scores (including significantly downregulated immune cell infiltration), consistent with our initial conclusion based on research results: immune microenvironment anti-tumor immunity would be beneficial for patient prognosis.

### 3.7. The Evaluation and Understanding of Model Performance

**Figure 8:**
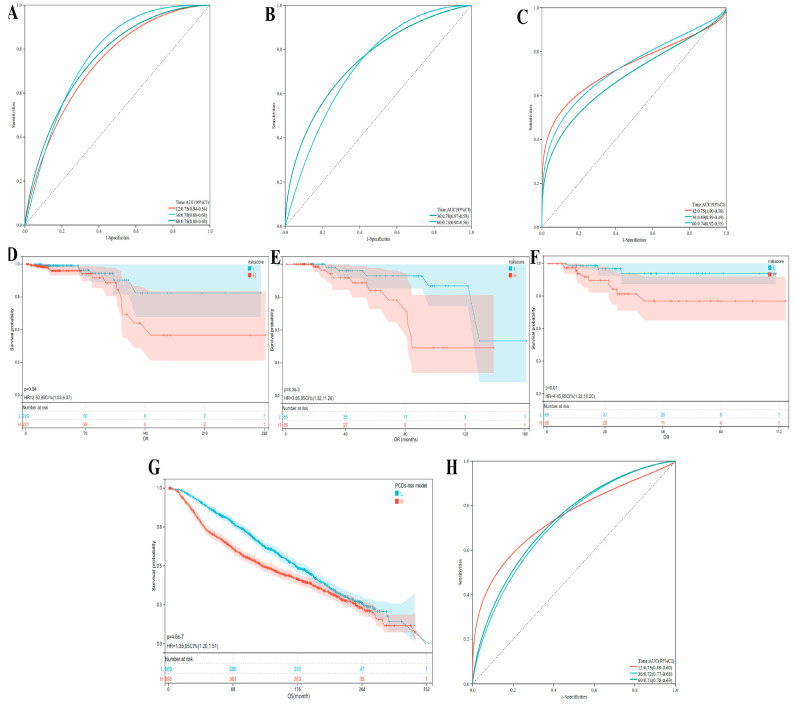
Validation of PCDs-related risk model based on TCGA and METABRIC breast cancer datasets. (**A**–**F**) ROC curve and K-M analysis based on this PCDs-related risk model, in (**A**,**D**) breast cancer Luminal-A, (**B**,**E**) Luminal-B, and (**C**,**F**) TNBC subtypes in TCGA-BCs cohort; (**G**,**H**) validation of PCDs-related risk model based on METABRIC breast cancer datasets, K-M analysis, and ROC curve in METABRIC cohort.

Furthermore, the PCDs-related risk model was validated in breast cancer Luminal-A, Luminal-B, and TNBC subtypes in TCGA-BCs cohort and METABRIC breast cancer cohort (Figure 8A–C,H). Utilizing the calculated PCDs risk score, we stratified BC patients in these above BC cohorts into PCDs-High and PCDs-Low groups. Our findings revealed a significant association between high PCDs risk and unfavorable clinical outcomes (Figure 8D–G).

**Figure 9:**
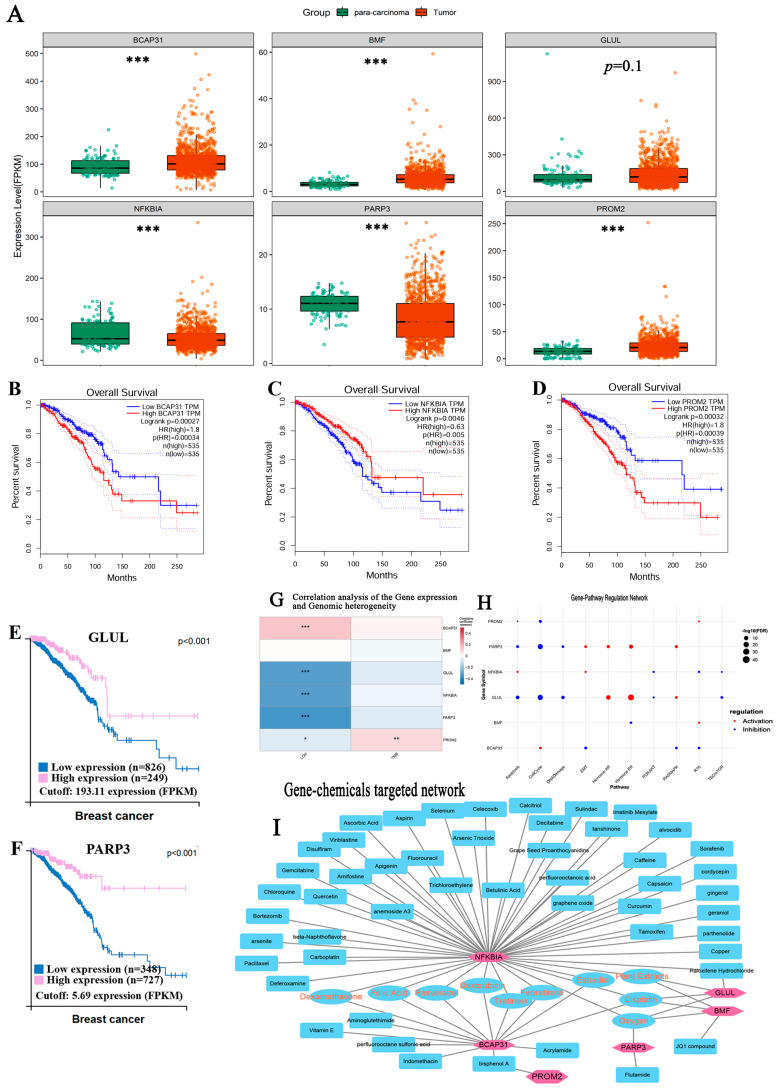
Prognostic and biological significance of involved gene signatures in PCDs-related risk model for breast cancer. (**A**) The gene expression levels of *BCAP31*, *BMF*, *GLUL*, NFKBIA, *PARP2,* and *PROM2* in breast cancer relative to Para cancerous tissue; (**B**–**D**) the prognostic value of *BCAP31*, NFKBIA, and *PROM2* for BC in Human Protein Atlas platform; (**E**,**F**) *GLUL* and *PARP3* were prognostic, and high expression is favorable in BC; (**G**) correlation coefficient analysis (Pearson) of the gene expression and genomic heterogeneity (TMB/LOH); (**H**) the gene-pathway regulation network via the GSCA platform showed that the expression of the gene signatures above could influence the activity of tumor-associated pathways. These data were based on the analysis of TCGA breast cancer dataset. (**I**) The gene and the potential targeted drugs based on the CTD database. (* *p* < 0.05, ** *p* < 0.01; *** *p* < 0.001).

In the PCDs risk model, we could observe *BCAP31* (B cell receptor associated protein 31), *BMF* (Bcl2 modifying factor) and *PROM2* (Prominin 2) significantly overexpressed in breast cancer compared with normal tissue, but *NFKBIA* (NFKB inhibitor alpha) and *PARP3* (Poly ADP-ribose polymerase family member 3) showed down-expressed level, with significant prognostic value for BC (shown in Figure 9A–D, and Figure S2A–F). Its expression level and prognostic value were consistent with the addition and subtraction directions of the calculation formula of the PCDs model: these above three genes, including *BACP31,* were risk factors, while *PARP3* and *NFKBIA* were protective factors. Based on the Human Protein Atlas datasets, *GLUL* and *PARP3* were genes associated with favorable prognosis in BC using the best cutoff separation, which divides the patients into two groups with 91%/86% 5-year survival for patients with high expression versus 79%/72% for patients with low expression, respectively (*p* < 0.001, Figure 9E,F). Meanwhile, their protein expression levels in breast cancer tissue showed a similar trend: BCAP31 displayed moderate cytoplasmic positivity in most malignant cells, especially in BC; BMF displayed moderate-to-strong cytoplasmic positivity; PROM2 displayed moderate-to-strong membranous and cytoplasmic positivity; GLUL was mainly negative in malignant cells, but moderate-to-strong staining also could be observed in some cases of BC and colorectal cancers: NFKBIA/PARP3 with weak cytoplasmic positivity (Figure S2G).

Our results indicated that the expression levels of *BCAP31*, *GLUL*, *NFKBIA*, *PARP3,* and *PROM2* were significantly correlated with genomic instability (Figure 9G). In addition, we constructed the gene-pathway regulation network via the GSCA platform, and the results indicated that the expression of above gene signatures could mainly influence the activity of apoptosis, cell cycle, epithelial–mesenchymal transition, Hormone AR, Hormone ER, RAS/MAPK, and RTK pathways (Figure 9H). We searched the CTD database for associated genes and the potential targeted drugs and found that *NFKBIA* is most closely associated with breast cancer progression, with a gene inference score (with BC) of 220.02, followed by *BCAP31* (inference score = 51.77), *BMF* (12.5), and *GLUL* (10.56). Many potential targeted drugs were identified based on the above genes, and resveratrol, dexamethasone, folic acid, tretinoin, fenretinide, doxorubicin, estradiol, and cisplatin might be important therapeutic drugs targeted by the above genes for BC (Figure 9I). These results indicated that *NFKBIA* was an important target gene for breast cancer therapy. It also deserves attention that drugs such as curcumin, bortezomib, sulindac, quercetin, celecoxib, arsenic trioxide, and parthenolide might exert anticancer effects by targeting *NFKBIA*.

## 4. Discussion

Breast cancer remains a significant global health challenge, representing the most frequently diagnosed cancer, and a leading cause of cancer-related mortality in women [14]. Despite more advancements in early detection and treatment, a significant proportion of patients experience disease recurrence and metastasis, highlighting the urgent need for a deeper understanding of the underlying mechanisms driving breast cancer progression and the development of novel therapeutic strategies to improve patient prognosis [15]. In this study, we focused on the impact of multiple programmed death patterns on the prognosis of breast cancer and explore the complex mechanism and intricate interplay behind them.

Our existing results supported that the apoptosis, pyroptosis, and autophagy activity of breast cancer tissues could significantly affect the prognosis of BC patients; that is, high-apoptosis, high-pyroptosis, and low-autophagy states could improve the BC prognosis underlying the mechanisms of the obvious immune-activated features and similar microenvironment states, via the more anti-tumor immune responses, specifically, mainly achieved through promoting macrophage polarization towards anti-tumorigenic M1 macrophages and high CD8+ T cells, Tfh and Tregs cells. Grivennikov et al. indicated that chronic inflammation within the TME could promote tumor growth and survival by suppressing apoptosis and promoting pro-survival autophagy [16], which was consistent with our research findings.

The TME comprises a complex network of cellular components, including immune cells, fibroblasts, endothelial cells, and the extracellular matrix, which dynamically interact with tumor cells and influence their behavior. Infiltrating immune cells were the focus of attention, such as CD8+ T cells, macrophages, Th1 cells, Tfh cells, and Treg cells, exhibit complex interactions with cancer cells and can influence PCD patterns [17]. CD8+ T cells, key effectors of anti-tumor immunity, can induce apoptosis in cancer cells through the release of cytotoxic molecules like perforin and granzyme B [18]. Macrophages, a heterogeneous population of immune cells, can exhibit both pro- and anti-tumor functions depending on their polarization state. M1 macrophages can promote tumor cell death through the production of pro-inflammatory cytokines and reactive oxygen species, while M2 macrophages can suppress anti-tumor immunity and promote tumor growth [19]. Th1 cells, a subset of CD4+ T cells, can enhance anti-tumor immunity by producing IFN-γ, which can promote apoptosis and inhibit angiogenesis. A study indicated sunitinib combined with Th1 cytokines promoted apoptosis in BC cells and suppresses tumor growth in a murine model of HER2(+) breast cancer [20]. Tfh cells, specialized in providing help to B cells, have been implicated in promoting anti-tumor immunity in certain contexts [21]. Treg cells, a subset of CD4+ T cells with immunosuppressive functions, can inhibit anti-tumor immunity and promote tumor growth by suppressing the activity of cytotoxic T cells and other immune cells [22]. A public single-cell sequencing dataset demonstrated increased infiltration of Tregs in TNBC tissues relative to normal breast tissue, and further, Huang et al. identified two Treg infiltration-associated clusters from TNBC that had different prognoses and sensitivities to drugs [23]. Understanding the intricate interplay between immune cells, PCD pathways, and the TME is crucial for developing effective immunotherapeutic strategies for breast cancer.

In the process of PCD patterns affecting the prognosis of breast cancer, besides immune response, ALK, VEGFA/VEGFR2, NF-κB, HIF-1, PI3K-Akt, MAPK, and JAK-STAT signaling pathways played an important role. Notably, ErbB, VEGF, TNF, and NF-κB pathways; chemokine signaling; focal adhesion; and leukocyte transendothelial migration associated with PCDs-related genes could explain the specificity of tumor metastasis mechanism in dead patients and further relate to the unfavorable prognosis of BC but with little impacts on patients with good prognosis.

In this study, we also explored the new tumor occurrence (Metastatic tumors and SPC) in BC during follow-up, an important risk factor that is easy to be ignored but would lead to severe poor prognosis. Some other studies indicated among women diagnosed with primary BC at or younger than age 40 years, their risk of a SPC is higher than that of women who are older when they develop a first primary BC [24]. We found tumor stage and immune phenotype (negative ER/PR/HER2 status) showed significant correlations with new tumor events. But its pathogenesis was mainly influenced by specific gene mutation spectrums, characterized by the increased loss of heterozygosity, and the obvious changes in mutation frequency in *TP53*, *CDH1*, *PIK3CA,* and *USH2A*, with no significant correlation with the TME and immune cell infiltration level.

Furthermore, by analyzing prognostic-related DEGs, we established a PCDs-related risk model using the LASSO method, with significant prognostic evaluation efficacy (for three/five-year survival, AUC = 0.76/0.76 in TCGA, N = 939; AUC = 0.72/0.74 in METABRIC, N = 1979). While the model achieved moderate AUC values (0.76 in TCGA; 0.74 in METABRIC), these values also indicated that the inherent heterogeneity of BC and the multifactorial nature of prognosis restricted the predictive performance of the model. Larger cohorts integrating multi-omics data (e.g., proteomics, spatial transcriptomics) may improve predictive accuracy. Notably, the model’s clinical utility lies in its ability to stratify high-risk patients (HR = 3.06, *p* < 0.001) for targeted monitoring. Based on this model, high-PCD-risk subtypes displayed the significant unfavorable prognosis, whose patients showed lower microenvironment matrix scores and immune scores (including significantly downregulated immune cell infiltration), suggesting that anti-tumor immunity acted as a dominant role in the interactive relation of PCD and TME. The model involved six gene signatures, *BCAP31*, *BMF*, *GLUL*, NFKBIA, *PARP2,* and *PROM2*. BCAP31, an endoplasmic reticulum protein, had been implicated in both apoptosis and autophagy regulation [25], with significantly high expression in BC and not conducive to prognosis. BMF, the protein encoded by proapoptotic the BCL-2-modifying factor (*BMF*) gene belongs to the BCL2 (B-cell lymphoma 2) family, was involved in the intrinsic apoptotic pathway [26]. Recent studies pointed out its important role in tumor drug resistance and enhancing chemosensitivity [27]. GLUL, an enzyme involved in glutamine metabolism, has been linked to autophagy regulation and tumor cell survival [28]. NFKBIA, an inhibitor of NF-κB signaling, plays a role in regulating apoptosis and inflammation [29]. PARP2, a DNA repair enzyme, is involved in both apoptosis and necroptosis [30]. Wang et al. found that the net effect of PARP inhibition on TAMs is a direct reprogramming and shift toward an anti-tumor activity, by mitigating the M2-inducing effects of IL-4, IL-10, and M-CSF [31]. Recent studies showed that the benefit of PARP inhibitors could extend beyond patients with germline BRCA1/2-associated metastatic breast cancer to somatic BRCA1/2 variants and germline PALB2 alterations [32]. The therapeutic effect of PARP inhibitors in other metastatic breast cancer lacked effective evidence. PROM2, a member of the PROM family of proteins, has been implicated in regulating mitochondrial function, apoptosis, and ferroptosis [33].

While our computational framework identified promising PCD-TME interaction axis, providing actionable insights for clinical practice translation, especially the TME phenotypes in the apoptosis/pyroptosis/necroptosis/autophagy score subtypes, further supports by a series of molecular biological experiments were required to enrich our investigation and to identify novel therapeutic targets that can effectively modulate PCD pathways and enhance anti-tumor immunity. The PCDs risk prognostic model (AUC = 0.76) not only stratifies high-risk patients but also highlights potential therapeutic targets (e.g., NFKBIA, PROM2, and PARP3) linked to immune response and genomic instability. And this model showed the potential and broad applicability for the risk prediction of various molecular subtypes in breast cancer.

This knowledge will pave the way for the development of innovative therapeutic strategies to improve patient outcomes in breast cancer. We believe that future research following up breast cancer patients, collecting tumor tissue samples for multi-omics research and biological experiments, and exploring the PCD-TME individual characteristics and their correlations with clinical treatment outcomes, metastasis/drug resistance progression, and prognosis could accelerate the development of precision medicine, which is also our important research direction.

## 5. Conclusions

By integrating multi-dimensional analyses of 13 PCD patterns, our study provides novel insights for understanding the PCD-TME-interaction-axis-driven breast cancer progression and prognosis and highlights that high apoptosis/pyroptosis and low autophagy states correlate with favorable prognosis, driven by enhanced anti-tumor immunity—specifically M1 macrophage polarization and CD8+ T cell activation. These immune-activated phenotypes underscore the therapeutic potential of modulating PCD pathways to reshape the TME. PCD-related genes promote metastasis and poor survival through VEGF/HIF-1/MAPK signaling and Th1/Th2 imbalance, while new tumor events (metastasis/secondary cancers) associate with TP53/CDH1 mutations and genomic instability. The LASSO 6-gene prognostic model, validated across multi-cohorts, can stratify high-risk BC patients (AUC = 0.76 in TCGA; 0.74 in METABRIC) and offer a clinically actionable tool for risk prediction. The high-risk subtypes by this model demonstrated unfavorable prognosis, characterized by suppressed immune infiltration and matrix remodeling. These findings advance our understanding of PCD-TME crosstalk in BC progression and offer a roadmap for integrating multi-omics data into precision oncology.

## Figures and Tables

**Table 1:** Clinical characteristics data for main subjects used in TCGA-BRCA datasets. (LNR, lymph node ratio, that is, the ratio of positive LNs to the total number of tested LNs, and we calculated the mean and standard deviation of the sample population; lymph nodes positive tests, number of patients detected with positive lymph nodes).

	Distant Metastasis (Metastatic Tumor)	New Primary Tumor	Control (Follow up Without New Neoplasm Occurrence)	*p*-Value
Age	57.3 ± 12.5	50.9 ± 12.0	58.7 ± 13.2	0.15
Survival status	Dead 69.4% (25/36)	Dead 40.0% (4/10)	Dead 11.3% (115/1020)	1.4e-24
T stage				0.75
T1	19.4% (7/36)	40% (4/10)	26.2% (267/1020)	
T2	55.6% (20/36)	60% (6/10)	58.1% (593/1020)	
T3	19.4% (7/36)	0	12.4% (126/1020)	
T4	5.6% (2/36)	0	3.3% (34/1020)	
N stage				0.03
N0	22.2% (8/36)	60% (6/10)	49.9% (509/1020)	
N1	44.4% (16/36)	30% (3/10)	32.4% (331/1020)	
N2	16.7% (6/36)	10% (1/10)	11.0% (112/1020)	
N3	16.7% (6/36)	0	6.7% (68/1020)	
M stage				0.02
M0	91.7% (33/36)	100%	98.1% (1001/1020)	
M1	8.3% (3/36)	0	1.9% (19/1020)	
Stage				7.9e-7
Stage I	5.9% (2/34)	22.2% (2/9)	17.5% (179/1020)	
Stage II	41.2% (14/34)	66.7% (6/9)	58.6% (598/1020)	
Stage III	44.1% (15/34)	11.1% (1/9)	22.5% (230/1020)	
Stage IV	8.8% (3/34)	0	1.9% (19/1020)	
Immune phenotype				
ER^−^	35.3% (12/34)	60.0% (6/10)	24.0% (216/990)	0.05
PR^−^	58.8% (20/34)	70.0% (7/10)	31.7% (313/987)	6.5e-3
HER2				0.03
amplifications	18.2% (4/22)	0	19.8% (181/913)	
HER2^−^	81.8% (18/22)	100.0% (6/6)	80.2% (732/913)	
TNBC	22.7% (5/22)	66.7% (4/6)	24.5% (224/913)	0.05
Lymph nodes				0.04
positive tests	76.3% (29/38)	44.4% (4/9)	49.8% (435/873)	
LNR	24.9 ± 29.1	6.7 ± 11.7	16.6 ± 27.0	
Margin status+	13.9% (5/36)	0	7.6% (72/945)	0.18

## Data Availability

The original contributions presented in the study are included in the article/Supplementary Material. Further inquiries can be directed to the corresponding authors.

## Supplementary Materials

The following supporting information can be downloaded at https://www.mdpi.com/article/10.3390/bioengineering12040420/s1: Table S1: PCD-related genes in our study. Figure S1: Complete Kaplan–Meier curves and the influence of PCD patterns for OS (A–L), PFS (M) and DSS (N) in BC. Figure S2: The expression of 6 gene signatures in breast cancer, utilizing GEPIA and Human Protein Atlas databases.

## Abbreviations

The following abbreviations are used in this manuscript:

PCD	Programmed cell death
BC	Breast cancer
TME	Tumor microenvironment
LASSO	Least Absolute Shrinkage and Selection Operator
WGCNA	weighted gene co-expression network analysis
TCGA	The Cancer Genome Atlas
ssGSEA	single-sample Gene Set Enrichment Analysis
KEGG	Kyoto Encyclopedia of Genes and Genomes
SPC	Second primary cancers
METABRIC	Molecular Taxonomy of Breast Cancer International Consortium
DEGs	Differential gene expression
TNBC	Triple-negative breast cancer
