# Supplementary figures and images for: Leveraging Diverse Cell-Death Patterns to Decipher the Interactive Relation of Unfavorable Outcome and Tumor Microenvironment in Breast Cancer
Source: Bioengineering (Basel). 2025 Apr 15;12(4):420. doi: 10.3390/bioengineering12040420 (PMC12024675; doi:10.3390/bioengineering12040420)

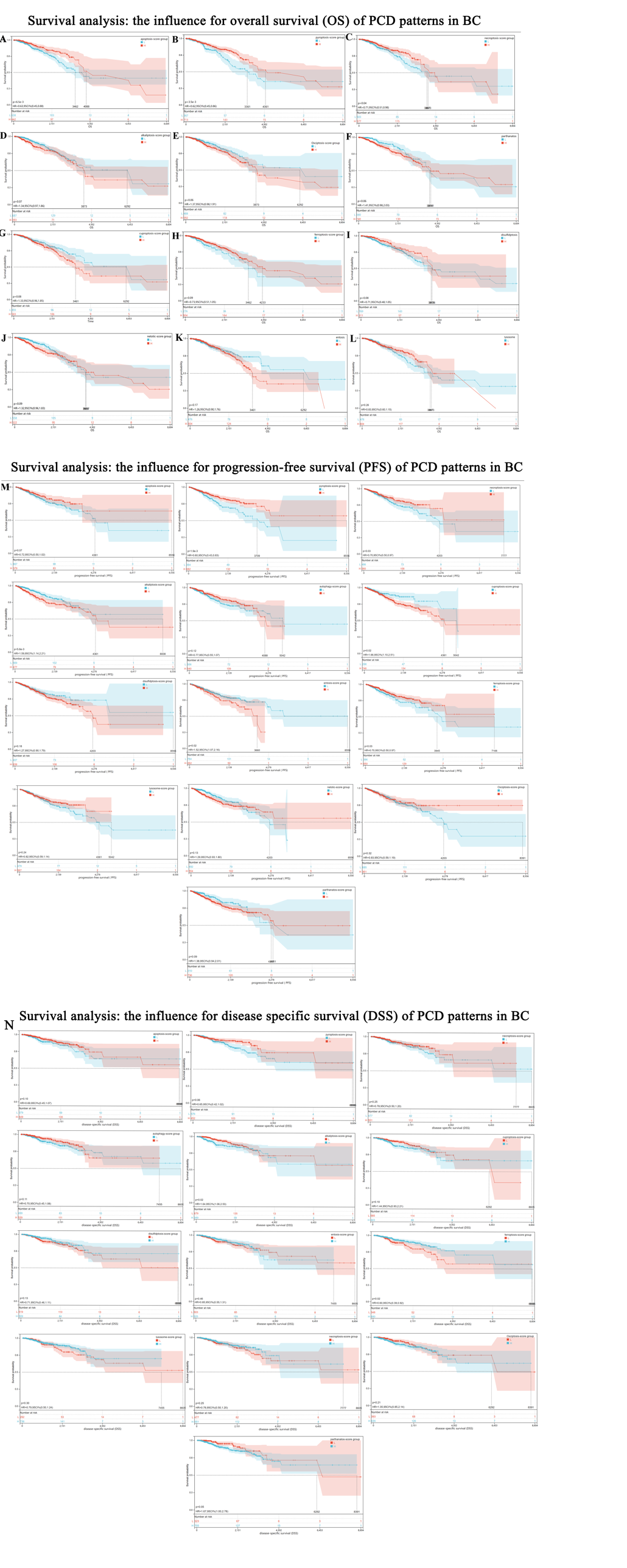

Supplement: Supplementary file 1 [file bioengineering-12-00420-s001.zip › figure S1.tif]

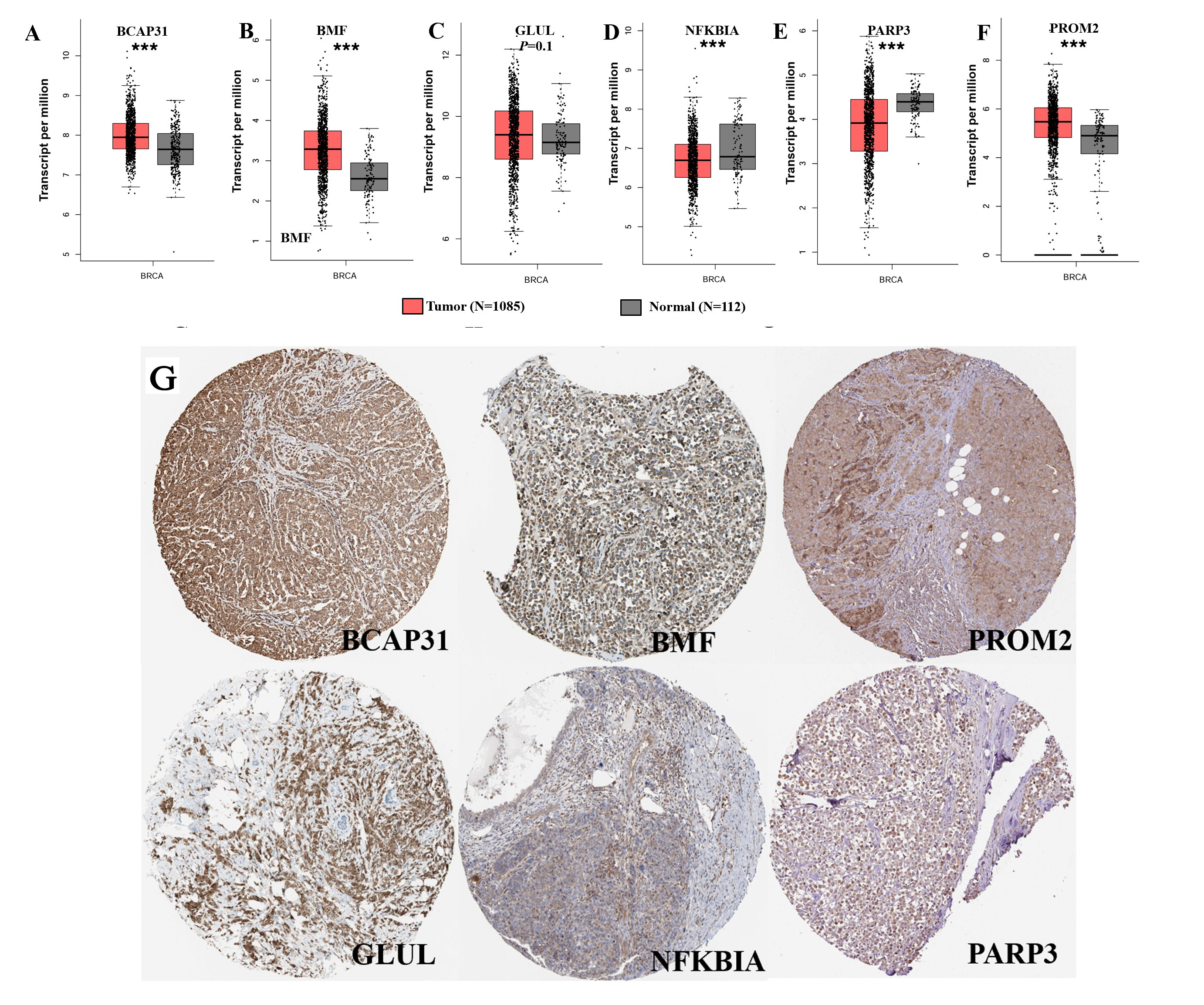

Supplement: Supplementary file 1 [file bioengineering-12-00420-s001.zip › figure S2.tif]
